# Accelerate the Electrolyte Perturbed-Chain Statistical Associating Fluid Theory–Density Functional Theory Calculation With the Chebyshev Pseudo-Spectral Collocation Method. Part II. Spherical Geometry and Anderson Mixing

**DOI:** 10.3389/fchem.2021.801551

**Published:** 2022-01-24

**Authors:** Yunhao Sun, Zhengxing Dai, Gulou Shen, Xiaohua Lu, Xiang Ling, Xiaoyan Ji

**Affiliations:** ^1^ Jiangsu Key Laboratory of Process Enhancement and New Energy Equipment Technology, School of Mechanical and Power Engineering, Nanjing Tech University, Nanjing, China; ^2^ Division of Energy Science/Energy Engineering, Luleå University of Technology, Luleå, Sweden; ^3^ State Key Laboratory of Materials-Oriented Chemical Engineering, Nanjing Tech University, Nanjing, China; ^4^ National and Local Joint Engineering Research Center for Deep Utilization Technology of Rock-Salt Resource, Faculty of Chemical Engineering, Huaiyin Institute of Technology, Huaian, China

**Keywords:** density functional theory, algorithm, ionic liquids, CO_2_, electrolyte perturbed-chain statistical associating fluid theory (ePC-SAFT)

## Abstract

To improve the efficiency of electrolyte perturbed-chain statistical associating fluid theory–density functional theory (ePC-SAFT-DFT) calculation of the confined system, in this work, first, the Chebyshev pseudo-spectral collocation method was extended to the spherical pores. Second, it was combined with the Anderson mixing algorithm to accelerate the iterative process. The results show that the Anderson mixing algorithm can reduce the computation time significantly. Finally, based on the accelerated ePC-SAFT-DFT program, a systematic study of the effects of the temperature, pressure, pore size, and pore shape on the CO_2_ solubilities in the ionic liquids (ILs) confined inside the silica nanopores was conducted. Based on the simulation results, to obtain high CO_2_ solubilities in the ILs confined in silica, a better option is to use the silica material with a narrow spherical pore, and the IL-anion should be selected specifically considering that it has a more significant impact on the absorption enhancement effect.

## 1 Introduction

Mitigating CO_2_ emission from fossil-fueled power plants as well as from transports has become an urgent and worldwide research topic, in which CO_2_ separation is often needed (MacDowell et al., 2010; Boot-Handford et al., 2014). Ionic liquids (ILs) are promising absorbents for CO_2_ separation due to their extremely low vapor pressure, high CO_2_ solubility, as well as low-energy usage for solvent regeneration (Brennecke and Gurkan, 2010; Ramdin et al., 2012; Zhang et al., 2012). However, the viscosity of pure ILs is relatively high compared with common organic solvents, causing a significant decrease in the mass and heat-transfer rates. Using supported ILs has been proposed as a promising solution for practical applications. This can take the advantage of high gas selectivity in ILs, and also, the high surface area of the supported materials can reduce the impact of high viscosity, improve the gas transfer, and hence increase the absorption rate (Zhang et al., 2006; Ren et al., 2012; Romanos et al., 2014).

Research has been conducted to address the confinement effect on the gas solubility in ILs via experiments and molecular simulations (Baltus et al., 2005; Ilconich et al., 2007; Ilconich et al., 2007; Zhang et al., 2010; Iarikov et al., 2011; Ren et al., 2012; Banu et al., 2013; Shi and Luebke, 2013; Romanos et al., 2014; Santos et al., 2014; Budhathoki et al., 2017; Shen and Hung, 2017). According to previous research, several factors will affect the CO_2_ solubility inside the confined ILs, for example, temperature, pressure, pore size, and shape of porous materials (Baltus et al., 2005; Zhang et al., 2006; Ilconich et al., 2007; Zhang et al., 2010; Iarikov et al., 2011; Ren et al., 2012; Banu et al., 2013; Shi and Luebke, 2013; Romanos et al., 2014; Santos et al., 2014; Budhathoki et al., 2017; Shen and Hung, 2017). However, to screen a suitable IL, optimizing the structure of supported material and operation conditions by experiment or molecular simulations is time and cost consuming, considering the fact that the huge number (10^18^) of possible ILs can be synthesized (Wasserscheid and Thomas, 2008), as well as the wide temperature and/or pressure range in applications. Therefore, it is desirable to develop a theoretical model to predict the properties of confined IL–CO_2_ systems.

The classical density functional theory (DFT) is considered as an efficient theoretical method for studying the confined properties (Tripathi and Chapman, 2005; Qiao et al., 2018; Shen et al., 2013; Xu et al., 2008; Sauer and Gross, 2017; Camacho Vergara et al., 2019). In addition, in our previous work (Ji et al., 2012; Ji et al., 2014; Shen et al., 2015; Ji and Held, 2016; Sun et al., 2019), electrolyte-perturbed-chain statistical associating fluid theory (ePC-SAFT) (Cameretti et al., 2005) has been developed to represent the thermodynamic properties of IL systems. Moreover, the developed ePC-SAFT has been combined with DFT (ePC-SAFT-DFT) to describe the properties of IL and CO_2_/IL confined in nanopores with acceptable results (Shen et al., 2018). Recently, in order to calculate the properties of the confined ILs with ePC-SAFT-DFT efficiently, the Chebyshev pseudo-spectral collocation method (Yatsyshin et al., 2012; Nold et al., 2017) was implemented to accelerate the ePC-SAFT-DFT calculation (Sun et al., 2021). However, only the slit-shaped and cylindrical pores have been considered previously, while for the spherical cavity, the corresponding method has not been available. In addition, an advanced iteration method is required for replacing the simple Picard iteration to accelerate ePC-SAFT-DFT calculation further. Anderson mixing is an elaborate iteration method that has been used in the work by Mairhofer and Gross (2017) and Shen et al. (2021), showing desirable performance in accelerating DFT computing. Therefore, replacing the Picard iteration with Anderson mixing can be an effective strategy.

In this work, the Chebyshev pseudo-spectral collocation method was extended to the spherical geometry, where an expression of 9-3 Lennard–Jones potential for the spherical cavity was derived. In addition, Anderson mixing was used to accelerate the ePC-SAFT-DFT calculation further. Based on the modified program, the CO_2_ solubility of ILs confined in the silica nanopores was chosen as the representative to conduct the investigation, considering silica is a promising supporting material for ILs (Shi and Luebke, 2013), and the effects of temperature, pressure, pore structures, and IL-ions were investigated systematically.

## 2 Theory

### 2.1 Electrolyte Perturbed-Chain Statistical Associating Fluid Theory-Density Functional Theory

According to DFT, in the presence of a solid surface, the grand potential Ω at grand canonical ensemble is given by the equation:

$$\Omega \left[\rho_{i}\left(r\right)\right]=A\left[\rho_{i}\left(r\right)\right]-\sum_{i}\int dr\prime \left[\rho_{i}\left(r\prime \right)(\mu_{i}-m_{i}V_{i,ext}\left(r\prime \right)\right]$$ (1)

where *A* is the Helmholtz free energy, $\rho_{i}\left(r\right)\;$ is the molecular density of component *i* at position ***r***, $\mu_{i}$ is the chemical potential, *m*_*i*_ is the number of segments in a chain for component *i*, and $V_{i,ext}\left(r\prime \right)$ is the nonelectrostatic external field acting on the segment of component *i*. The Helmholtz free energy *A* in ePC-SAFT-DFT for a system can be expressed as (Qiao et al., 2018; Shen et al., 2018):

$$A\left[\rho_{i}\left(r\right)\right]=A^{id}\left[\rho_{i}\left(r\right)\right]+A^{hs}\left[\rho_{i}\left(r\right)\right]+A^{chain}\left[\rho_{i}\left(r\right)\right]+A^{disp}\left[\rho_{i}\left(r\right)\right]+A^{ion}\left[\rho_{i}\left(r\right)\right]$$ (2)

where $A^{id}$ is the ideal free energy, $A^{hs}$ , $A^{chain}$ , $A^{disp}$ , and $A^{ion}$ are the excess free energies due to hard-sphere repulsions, chain connectivity, dispersive, and electrostatic interactions, respectively. The model performance has been verified in a previous work (Shen et al., 2018), where the model predictions in the density profiles of ionic fluids in the charged pores were compared with the molecular simulation results with high consistency.

The details of $A^{hs}$ , $A^{chain}$ , and $A^{disp}$ have been described elsewhere ( $A^{hs}$ : Barker and Henderson, 1967; Rosenfeld, 1989; Roth et al., 2002; Yu and Wu, 2002a; Yu and Wu, 2002b; $A^{chain}:$ Tripathi and Chapman, 2005; $A^{disp}:$ Shen et al., 2013). The $A^{ion}$ is composed of two terms, the Coulomb term ( $A^{col}$ ) and the Debye–Hückel term $\left(A^{DH}\right)$ . $\;A^{DH}$ accounts for the short-range electrostatic interaction caused by the inhomogeneous distribution at the nearby region of ions, which is based on the Debye–Hückel (DH) theory. $A^{col}$ accounts for the Helmholtz free energy caused by the long-range electrostatic interaction due to the unsymmetrical distribution of cation and anion. The expressions of $A^{col}$ and $A^{DH}$ can be found in Li and Wu (2006) and Shen et al. (2018), respectively. All these terms are involved in the ePC-SAFT-DFT calculations for the IL–CO_2_ systems.

Minimization of the grand potential with respect to the density profile of component *i* yields the following Euler–Lagrange equation:

$$\frac{\delta \Omega \left[\rho \left(r\right)\right]}{\delta \rho_{i}\left(r\prime \right)}=\sum_{c}\frac{\delta A^{c}\left[\rho \left(r\right)\right]}{\delta \rho_{i}\left(r\prime \right)}+q_{i}\psi \left(r\prime \right)-\left(\mu_{i}-m_{i}V_{i,ext}\left(r\prime \right)\right)=0\left(c=id,\;hs,\;chain,\;disp,\;DH\right)$$ (3)

where *q*_*i*_ is the charge of component *i*, and ψ(r) is the mean electric potential at position ***r***, which can be obtained by solving the Poisson’s equation (Li and Wu, 2006). The expressions of functional derivatives $\frac{\delta A^{c}\left[\rho \left(r\right)\right]}{\delta \rho_{i}\left(r\right)}$ are described in Rosenfeld et al. (1997), Gross (2009), Roth (2010), and Sun et al. (2021).

### 2.2 Evaluation of the Convolution-Like Integrals for Spherical Cavity

The expressions of the functional derivatives $\frac{\delta A^{c}\left[\rho \left(r\right)\right]}{\delta \rho_{i}\left(r\right)}$ are described in Rosenfeld et al. (1997), Gross (2009), Roth (2010), and Sun et al. (2021). All the convolution-like integrals involved in ePC-SAFT-DFT calculation can be classified as:

$$\{\begin{array}{c} I_{1}\left(r\right)=\int dr\prime f\left(r\prime \right)\delta \left(R_{c}-|r-r\prime |\right)\\ I_{2}\left(r\right)=\int dr\prime f\left(r\prime \right)\frac{\vec{r}-\vec{r}\prime }{\left|\vec{r}-r\prime \right|}\delta \left(R_{c}-|r-r\prime |\right)\\ I_{3}\left(r\right)=\int dr\prime f\left(r\prime \right)\theta \left(R_{c}-|r-r\prime |\right) \end{array}$$ (4a)

where *R*_*c*_ represents the weighting distances, which are:

$$\{\begin{array}{c} R_{c}=\frac{d_{i}}{2},\;\;\;for\;the\;hard\;sphere\;and\;association\;term\\ R_{c}=d_{i},\;\;\;\;\;\;\;\;\;\;\;\;\;\;\;\;\;\;\;\;\;\;\;\;\;\;for\;the\;chain\;term\\ R_{c}=\lambda \sigma_{i},\;\;\;\;\;\;\;\;\;\;\;\;\;\;for\;the\;dispersion\;term\\ R_{c}=\frac{d_{i,DH}}{2},\;\;\;\;\;\;\;\;\;\;\;\;\;\;\;\;\;\;\;\;\;\;\;\;\;\;\;for\;the\;DH\;term \end{array}$$ (4b)

In Eq. 4b, θ is the Heaviside step function, and δ is the Dirac delta function. The $\sigma_{i}$ and $d_{i}$ in Eq. 4b represent the temperature-independent and -dependent hard sphere segment diameter (Barker and Henderson, 1967), respectively. The $d_{i,DH}$ is the weighting distance for the Debye–Hückel term, which can be referred to in Shen et al. (2018). The λ was set as 1.5 in order to be consistent with the bulk PC-SAFT model (Gross and Sadowski, 2001). The convolution integrals in Eq. 7a map f(r′) to I(r) with these integral kernels.

For spherical cavities, f(r′) and I(r) only vary with the radial direction, and the analytic expressions of I(r) reads (Groh and Mulder, 2000):

$$\{\begin{array}{c} \;I_{1}\left(r\right)=\frac{2\pi R_{c}}{r}\overset{r+R_{c}}{\underset{\left|r-R_{c}\right|}{\int }}\mathrm{dr}\prime f\left(r\prime \right)r\prime \\ I_{2}\left(r\right)=\frac{\pi }{r^{2}}{\vec{e}}_{r}\overset{r+R_{c}}{\underset{\left|r-R_{c}\right|}{\int }}\mathrm{dr}\prime f\left(r\prime \right)r\prime {(r^{2}-r\prime }^{2}+R_{c}^{2})\\ I_{3}\left(r\right)=\frac{\pi }{r}\int_{\left|r-R_{c}\right|}^{r+R_{c}}\mathrm{dr}\prime f\left(r\prime \right)r\prime \left[R_{c}^{2}-{\left(r-r\prime \right)}^{2}\right]+4\pi \theta \left(R_{c}-r\right)\overset{R_{c}-r}{\underset{0}{\int }}\mathrm{dr}\prime f\left(r\prime \right)r\prime^{2} \end{array}$$ (5)

### 2.3 Evaluation of Mean Electric Potential Distribution Inside Spherical Cavity

In the spherical symmetric distribution, the Poisson’s equation reduces to:

$$\frac{d^{2}\left[r^{2}\psi \left(r\right)\right]}{r^{2}dr^{2}}=-\frac{1}{\varepsilon_{0}\varepsilon_{r}}q\left(r\right)$$ (6)

where q(r) is the total charge at position *r*.

For a spherical cavity with diameter *R*, by solving Eq. 6 with the boundary conditions $\psi \left(R\right)=\psi^{w}$ and $\frac{d\psi \left(0\right)}{dr}=0$ , the expression for the mean electric potential reads:

$$\psi \left(r\right)=\psi^{w}+\frac{1}{\varepsilon_{0}\varepsilon_{r}}\left[\frac{R-r}{Rr}\int_{0}^{r}dr\prime q\left(r\prime \right)r\prime^{2}+\int_{r}^{R}dr^{\prime }\frac{R-r\prime }{R}q\left(r\prime \right)r\prime \right]$$ (7)

According to Eq. 7, the boundary electric potential $\psi^{w}$ is required, which corresponds to a specific charge density on the internal surface of a nanopore. This value can be obtained *via* “trial and error” until the charge of ions in the nanopore is equal in magnitude to that with the opposite sign on the internal surface of the nanopore.ü

### 2.4 Chebyshev Pseudo-Spectral Collocation Method

The Chebyshev pseudo-spectral collocation method for DFT modeling was developed by Yatsyshin et al. (2012). In the Chebyshev pseudo-spectral collocation method, for one-dimensional DFT calculation with an *N*-point discretization scheme, the density or the weighted density profile over the whole computation domain is determined from the density or the weighted density at a prescribed set of collocation points {*z*_*k*_}, *k* = 1,2 … , *N* using the barycentric form (Baltensperger, 2002; Berrut and Trefethen, 2004):

$$\rho \left(z\right)=\frac{\sum_{1}^{\prime N}\frac{{\left(-1\right)}^{k}}{z-z_{k}}\rho \left(k\right)}{\sum_{1}^{\prime N}\frac{{\left(-1\right)}^{k}}{z-z_{k}}}$$ (8)

where the primes indicate that the first and last terms in the sums are divided by 2. The collocation points (*z*_*k*_) can be obtained by a conformal map from the Chebyshev collocation points (*x*_*k*_) (Baltensperger, 2002; Berrut and Trefethen, 2004):

$$x_{k}=cos\frac{\left(k-1\right)\pi }{N-1}$$ (9)

The integrals associated with the DFT calculation can be evaluated with the Clenshaw–Curtis quadrature (Clenshaw and Curtis, 1960).

### 2.5 Implementation of Chebyshev Pseudo-Spectral Collocation Method in Electrolyte Perturbed-Chain Statistical Associating Fluid Theory-Density Functional Theory for Spherical Cavity

In the ePC-SAFT-DFT calculation, three domains need to be discretized for interpolation. The first domain is for the density profile, the second one is for the weighted density function profiles in the hard-sphere term, and the third one is for the weighted density function profile in the Debye–Hückel term.

For the spherical cavity with diameter *R*, we defined a diameter *R′* as:

$$R\prime =R-\sigma_{s}$$ (10)

**FIGURE 1:**
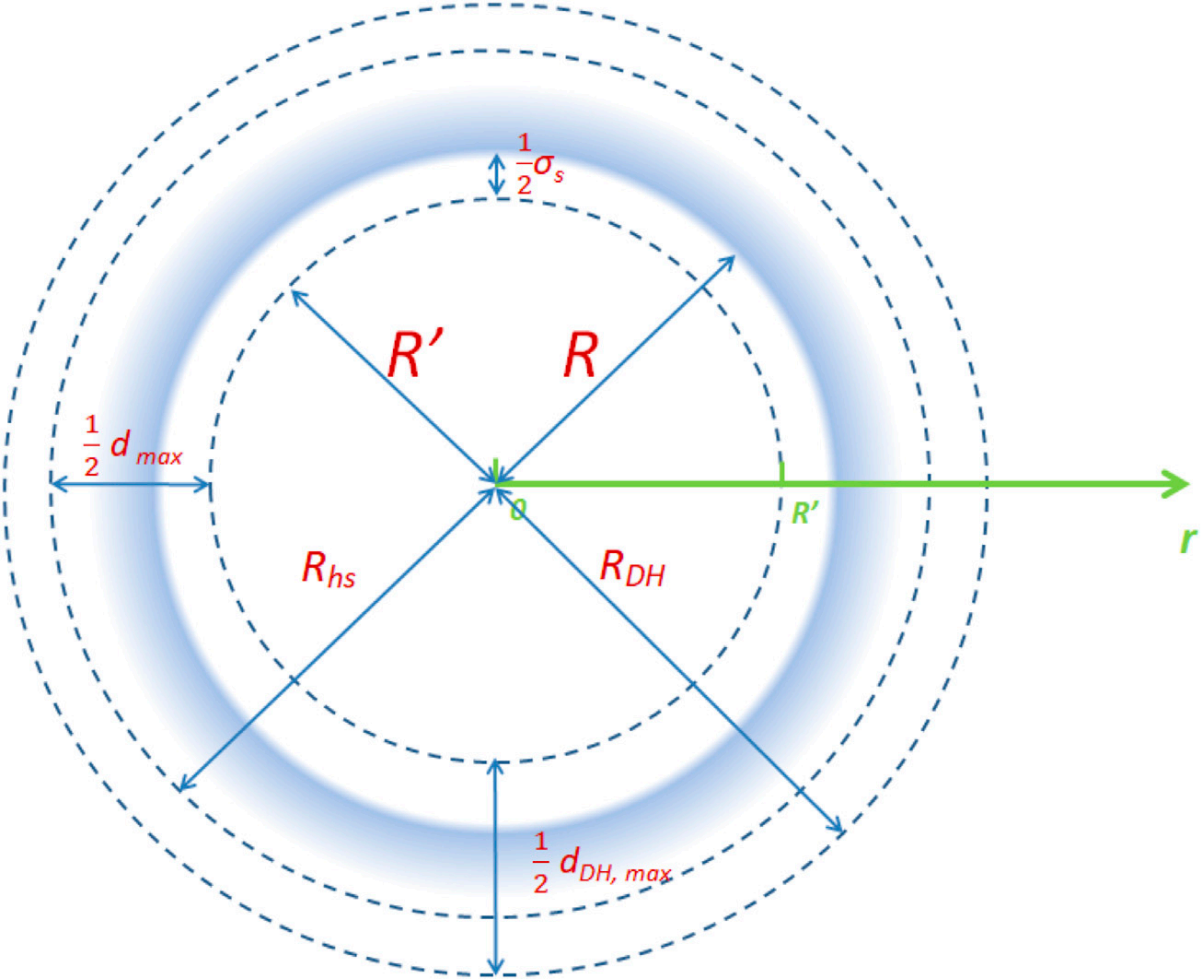
Schematic diagram of the domains considered in a spherical cavity.

The density profile is considered in the domain (0, *R'*) based on the coordinate system illustrated in Figure 1. In other words, this domain is discretized for interpolating the density profile function. In general, the density profile vibrates dramatically near the wall, which implies that more collocation points are required in these regions compared with the middle of the nanopore*.* In this work, the conformal map proposed by Bayliss and Turkel was used to map the Chebyshev collocation points $\left\{x_{k}\right\}$ to domain (0, *R'*) (Bayliss and Turkel, 1992):

$$z_{k}=\frac{R\prime }{2}\left\{\beta +\frac{1}{\alpha }\tan\left[\lambda \left(x_{k}-\mu \right)\right]\right\}+\frac{R\prime }{2}$$ (11a)

The λ and μ in Eq. 11a read:

$$\{\begin{array}{c} \lambda =\frac{\gamma +\eta }{2}\\ \mu =\frac{\gamma -\eta }{\gamma +\eta } \end{array}$$ (11b)

where:

$$\{\begin{array}{c} \gamma =\arctan\left[\alpha \left(1+\beta \right)\right]\\ \eta =\arctan\left[\alpha \left(1-\beta \right)\right] \end{array}$$ (11c)

Equation 11a maps the Chebyshev points in the transformed coordinate into the points that cluster in the physical coordinate near $\frac{R\prime }{2}\left(\beta +1\right)$ with a density that is large when α is large. In this work, the β was set as:

$$\beta =1-\frac{\bar{d}}{R\prime }$$ (12)

where $\bar{d}$ is the mean diameter of all components.

Therefore, the collocation points are clustered in the physical coordinate near ( $R\prime -\frac{\bar{d}}{2}$ ), where high peaks have occurred in the nearby region. The α was set as 2 in this work. Similar approaches were used for the discretization of another two domains, i.e., the domains for weighted density function profiles of hard-sphere term (0, *R*_*hs*_) and Debye–Hückel term (0, *R*_*DH*_).

#### 2.5.1 Evaluation of Convolution-Like Integral

As pointed out by Yatsyshin et al. (2012), the maps (Eq. 4) involved in DFT calculation can be carried out by matrix–vector products with discrete data based on interpolation (Eq. 8) and Clenshaw–Curtis quadrature. In the spherical cavities, for a map with original space discretized into *N*_*2*_ points and the image space discretized into *N*_*1*_ points, the map can be represented by *N*_*1*_ × *N*_*2*_ matrix (Yatsyshin et al., 2012):

$$J_{\mathrm{ij}}=\{\begin{array}{c} \frac{J_{\mathrm{ij}}^{\prime }}{2}\;\;\;\;j=1,N_{2}\\ J_{\mathrm{ij}}^{\prime }\;\;\;j\in \left[2,N_{2}-1\right] \end{array}$$ (13a)

with

$$J_{\mathrm{ij}}^{\prime }=\frac{\left(b_{i}-a_{i}\right){\left(-1\right)}^{j}}{2}\sum_{q=1}^{M}\frac{\frac{{\hat{w}}_{q}W\left(z_{i}-y_{qi}\right)}{y_{qi}-z_{j}}}{\sum_{m=1}^{N_{2}}\frac{{\left(-1\right)}^{m}}{y_{qi}-z_{m}}}$$ (13b)

where *a*_*i*_ and *b*_*i*_ are the lower and upper limits of integral for the *i*th discrete point, *W* is the corresponding integral kernel in Eq. 5, and $y_{qi}$ is the *q*th Chebyshev grid of the *i*th discrete point in the image space:

$$y_{qi}=a_{i}+\left(b_{i}-a_{i}\right)x_{q}$$ (13c)

${\hat{w}}_{q}$ is the corresponding Clenshaw–Curtis weight, and the values at *M* Chebyshev grids are used to evaluate each integral. In this work, *M* was set as 45.

For the singularities in the expression of Eq. 13b (i.e., $y_{qi}=z_{k}$ ), the corresponding elements can be evaluated based on the following expression (Sun et al., 2021):

$$\underset{y_{qi}\to Z_{k}}{\lim}\frac{\frac{{\hat{w}}_{q}}{y_{qi}-Z_{j}}}{\sum_{m=1}^{N_{2}}\frac{{\left(-1\right)}^{m}}{y_{qi}-Z_{m}}}=\{\begin{array}{c} 0\;\;\;\;\;\;\;\;\;\;\;\;\;\;\;\;\;j\;\neq k\\ 2\;\;\;\;\;\;\;\;\;\;j=k=1\\ 2{\left(-1\right)}^{N_{2}}\;\;\;\;\;j=k=N_{2}\\ \;{\left(-1\right)}^{k}\;\;1<\;j=k<N_{2} \end{array}\;\;\;\;\;\;\;$$ (13d)

#### 2.5.2 Evaluation of the Mean Electric Potential

Equation 7 can be represented with matrix–vector products with the discrete data:

$$\vec{\psi }=\frac{1}{\varepsilon_{0}\varepsilon_{r}}\left(D_{1}\cdot \vec{q}+D_{2}\cdot \vec{q}\right)+\psi^{w}$$ (14a)

where $\vec{\psi }$ and $\vec{q}$ are composed of ψ and *q* at the position of collocation points, respectively.

The two *N *× *N* matrixes $D_{1}$ and $D_{2}$ read:

$$\{\begin{array}{c} D_{1}=L\prime \cdot Q_{1}\\ D_{2}=U\prime \cdot Q_{2} \end{array}$$ (14b)

where L′ and U′ are composed of:

$$\{\begin{array}{c} L\prime =\left[\begin{array}{c} 0\\ L \end{array}\right]\\ U\prime =\left[\begin{array}{c} U\\ 0 \end{array}\right] \end{array}$$ (14c)

where L and U are the lower and upper triangular matrices with all elements being unity, respectively. The elements in the (*N-1*) × *N* matrix $Q_{1}$ read:

$$Q_{1,\mathrm{ij}}=\{\begin{array}{c} \frac{Q_{1,\mathrm{ij}}^{\prime }}{2}\;\;\;\;j=1,N\\ Q_{1,\mathrm{ij}}^{\prime }\;\;\;j\in \left[2,N-1\right] \end{array}$$ (14d)

with

$$Q\prime_{1\mathrm{,ij}}=\frac{\left(R\prime -Z_{i+1}\right)\left(b_{i}-a_{i}\right){\left(-1\right)}^{j}}{2R\prime Z_{i+1}}\sum_{q=1}^{M}\frac{\frac{{\hat{w}}_{q}y_{qi}^{2}}{y_{qi}-Z_{j}}}{\sum_{m=1}^{\prime N_{2}}\frac{{\left(-1\right)}^{m}}{y_{qi}-Z_{m}}}$$ (14e)

The elements in (*N-1*) × *N* $Q_{2}$ read:

$$Q_{2\mathrm{,ij}}=\{\begin{array}{c} \frac{Q\prime_{2\mathrm{,ij}}}{2}\;\;\;\;j=N\\ Q\prime_{2\mathrm{,ij}}\;\;\;j\in \left[1,N-1\right] \end{array}$$ (14f)

with

$$Q\prime_{2\mathrm{,ij}}=\frac{\left(b_{i}-a_{i}\right){\left(-1\right)}^{j}}{2R^{\prime }}\sum_{q=1}^{M}\frac{\frac{{\hat{w}}_{q}\left(R\prime -y_{qi}\right)y_{qi}}{y_{qi}-z_{j}}}{\sum_{m=1}^{N_{2}}\frac{{\left(-1\right)}^{m}}{y_{qi}-Z_{m}}}\;$$ (14g)

where

$$\{\begin{array}{c} a_{i}=z_{i}\\ b_{i}=z_{i+1} \end{array}$$ (14h)

$y_{qi}$ can be evaluated with Eq. 13c with the *a*_*i*_ and *b*_*i*_ defined in Eq. 14h. The singularities in equations Eq. 14e and Eq. 14g can also be evaluated based on Eq. 13d.

$Q_{1}$ and $Q_{2}$ are the linear operators implementing the piecewise integrations between two adjacent collocation points, while L′ and U′ are used to sum up the results of these piecewise integrations for obtaining the final integration results of Eq. 7.

### 2.6 Solving Euler–Lagrange Equation With Anderson Mixing

In the Chebyshev pseudo-spectral collocation method, the Euler–Lagrange equation (Eq. 3) is transformed into a vector equation that can be solved numerically. In our previous work, the Picard iteration was used. In this work, the Anderson mixing is implemented to solve Eq. 3 iteratively (Anderson, 1965). In the Anderson mixing, the Euler–Lagrange equation can be rewritten as:

$$\rho_{i}\left(z\right)=\rho_{i,b}\exp\left(-\frac{V_{i,ext}\left(z\right)}{kT}+\frac{\mu_{i}^{res}+\sum_{c}\frac{\delta A^{c}}{\delta \rho_{i}}\left(z\right)+q_{i}\psi \left(z\right)}{kTm_{i}}\right)=g\left[\rho_{i},z\right]\left(c=\;hs,\;chain,\;disp,\;DH\right)\;$$ (15a)

where *k* represents the Boltzmann constant, $\rho_{i,b}$ represents the bulk phase density of component *i*, and the superscript *res* represents the residual quantities.

Eq. 15a can be solved iteratively by (Anderson, 1965):

$$\rho_{i}^{k+1}(z)=(1-S)\sum_{j=0}^{m^{k}}\gamma_{j}^{k}\rho_{i}^{k-m^{k}+j}\left(z\right)+S\sum_{j=0}^{m^{k}}\gamma_{j}^{k}g[\rho_{i}^{k-m^{k}+j},z]$$ (15b)

where *S* is the relaxing factor, $m^{k}=\text{min}\left(m,k\right)$ , *m* was set as 50 in this work, the subscript *i* represents the *i*th component, and $\gamma_{j}^{k}$ is determined by:

$$\gamma_{j}^{k}=\underset{\gamma_{0}^{k},\ldots \gamma_{m^{k}}^{k}}{\min}\sqrt{\sum_{i}\sum_{z}\sum_{j=0}^{m^{k}}\gamma_{j}^{k2}{\left\{g\left[\rho_{i}^{k-m^{k}+j},z\right]-\rho_{i}^{k-m^{k}+j}\left(z\right)\right\}}^{2}}\;s.t.\sum_{j=0}^{m^{k}}\gamma_{j}^{k}=1$$ (15c)

The constrained optimization can be transformed to unconstrained optimization (Fang and Saad, 2008; Walker and Ni, 2011):

$$\omega_{j}^{k}=\underset{\omega_{0}^{k},\ldots \omega_{m^{k}-1}^{k}}{\min}\sqrt{\sum_{i}\sum_{z}\{{ℱ}_{i}^{k}\left(z\right)-\sum_{j=0}^{m^{k}-1}\omega_{j}^{k}[{ℱ}_{i}^{k-m^{k}+j+1}\left(z\right)-{ℱ}_{i}^{k-m^{k}+j}\left(z\right)]{\}}^{2}}$$ (15d)

where

$$\omega_{j}^{k}=\{\begin{array}{c} \gamma_{j}^{k}-\gamma_{j-1}^{k}\;\;\;j>1\\ \gamma_{0}^{k}\;\;\;\;\;j=0 \end{array}\;and\;{ℱ}_{i}^{n}\left(z\right)=g\left[\rho_{i}^{n},z\right]-\rho_{i}^{n}\left(z\right)$$ (15e)

The successive optimization (Eq. 15d) can be solved efficiently by the updating QR factorization, and the necessary Matlab code is given in Walker (2011). In order to avoid divergence, *Ns* steps of Picard iterations can be performed at the beginning and then switched to the Anderson mixing procedure.

### 2.7 General Scheme Combined With Anderson Mixing

**SCHEME 1:**
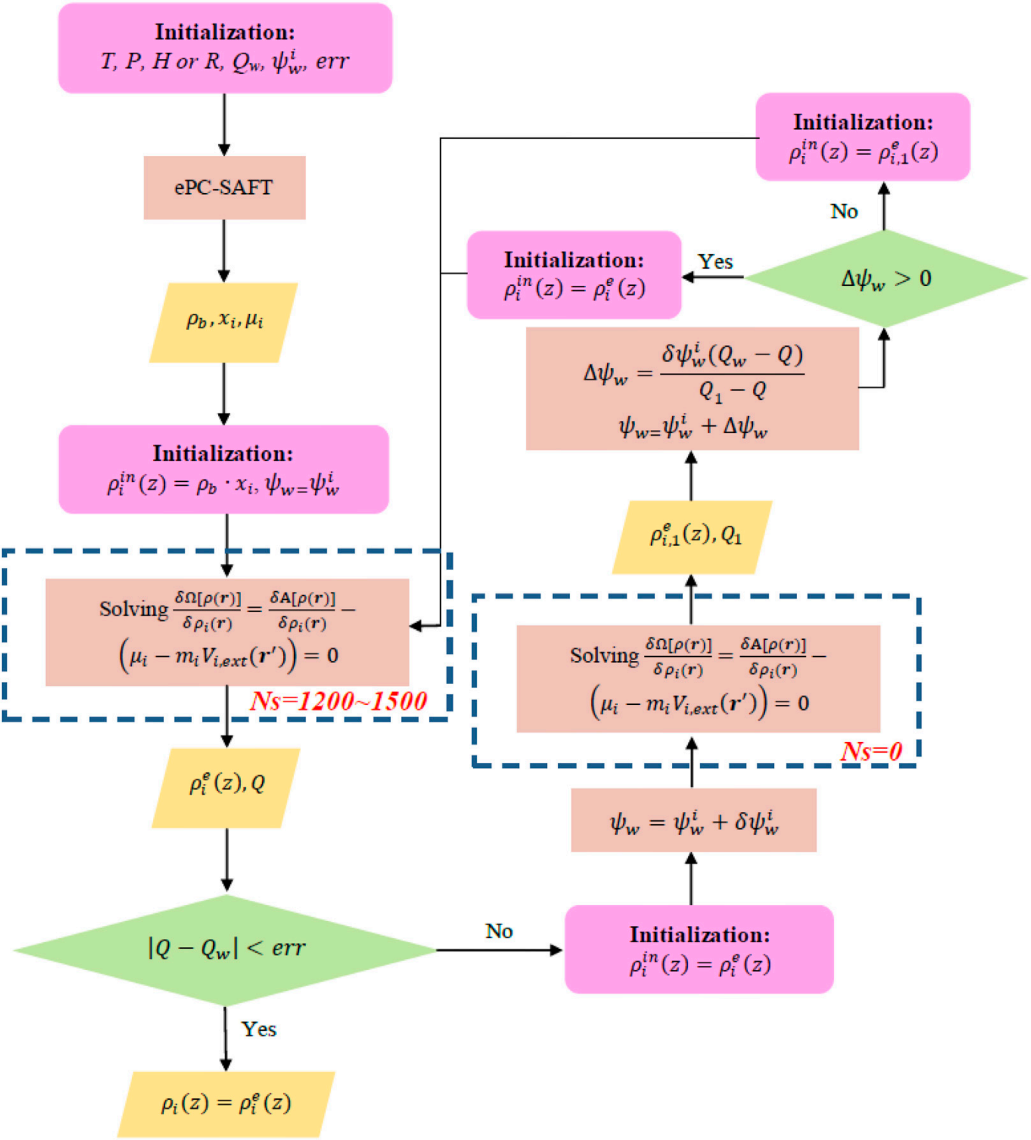
Calculating the density profile of ionic liquid (IL)–CO_2_ system inside nanopores with specific surface charge $Q_{w}$ using the general scheme combined with the Anderson mixing.

A general scheme for calculating the density profile of confined systems with ionic contribution was proposed in Sun et al. (2021), as illustrated in Scheme 1. In a loop of Scheme 1, the Euler–Lagrange equation needs to be solved twice. For the first time, solving the Euler–Lagrange equation in a loop, *Ns* (steps of Picard iteration performed before the Anderson mixing procedure) can be set between 1,200 and 1,500, while for the second time, due to the initial guess of density profile being not far away from the equilibrium density profile, we do not perform Picard iterations prior to the Anderson mixing procedure. (i.e., *Ns *= 0).

In Scheme 1, *T*, *p*, $\mu_{i}$ , and *x*_*i*_ refer to those in the bulk phase, which were obtained with ePC-SAFT. *H* (or *R*) and *Q*_*w*_ are the features of nanopores. $\rho^{in}$ represents the initial guess of the density profile, and $\rho^{e}$ represents the calculated equilibrium density profile with the boundary electric potential $\psi_{w}$ . The charge absorbed per area of nanopore *Q* can be computed with:

$$Q=\underset{i}{\sum }q_{i}{\bar{\rho }}_{i}$$ (16a)

where the amount of component *i* adsorbed per surface area ${\bar{\rho }}_{i}$ for the slit-like pores can be evaluated by:

$${\bar{\rho }}_{i}=\frac{\int_{0}^{H}dz\rho_{i}\left(z\right)}{2}$$ (16b)

for the cylindrical pores:

$${\bar{\rho }}_{i}=\frac{\int_{0}^{R}drr\rho_{i}\left(r\right)}{R}$$ (16c)

and for the spherical cavities:

$${\bar{\rho }}_{i}=\frac{\int_{0}^{R}drr^{2}\rho_{i}\left(r\right)}{R^{2}}$$ (16d)

### 2.8 Model Ionic Liquid–CO_2_ Confined in Nanopores

Following ePC-SAFT for the ILs in the bulk systems (Ji et al., 2012), an ionic liquid molecule is composed of one IL cation and one IL anion. Each individual IL ion was modeled as a nonspherical species with repulsion, dispersive attraction, and Coulomb interactions. The 9-3 Lennard–Jones potential was used to represent the nonelectrostatic interaction between silica and fluid. The nanopore was modeled as an infinitely large slit, infinitely long cylinder, or spherical cavity. The 9-3 Lennard–Jones potential for large slit and infinitely long cylinder have already been presented in the literature (Fitzgerald et al., 2003; Siderius and Gelb, 2011; Lee, 2016), while for a spherical cavity with diameter *R*, the 9-3 Lennard–Jones potential can be obtained from:

$$\begin{array}{l} U_{s,9-3,i}\left(r\right)=\rho_{atom}\int_{0}^{2\pi }d\varphi \int_{0}^{\pi }d\theta \int_{R}^{+\infty }dr\prime 4\varepsilon_{si}\left[{\left(\frac{\sigma_{si}}{\sqrt{r^{\prime 2}+r^{2}-2rr\prime cos\theta }}\right)}^{12}-{\left(\frac{\sigma_{si}}{\sqrt{r^{\prime 2}+r^{2}-2rr\prime cos\theta }}\right)}^{6}\right]r\prime^{2}sin\theta \\ =\{\begin{array}{c} 2\pi \rho_{atom}\sigma_{si}^{3}\varepsilon_{si}\left\{\frac{1}{180}\left[\frac{9R-r}{r{\left(R-r\right)}^{9}}-\frac{9R+r}{r{\left(R+r\right)}^{9}}\right]\sigma_{si}^{9}-\frac{8}{3}\frac{R^{3}}{{\left(R^{2}-r^{2}\right)}^{3}}\sigma_{si}^{3}\right\}\;\;r>0\\ 2\pi \rho_{atom}\sigma_{si}^{3}\varepsilon_{si}\left(\frac{8}{9R^{9}}\sigma_{si}^{9}-\frac{8}{3R^{3}}\sigma_{si}^{3}\right)r=0 \end{array} \end{array}$$ (17a)

where *r* is the distance of the fluid molecule from the center of the spherical cavity, $\rho_{atom}$ is the solid atom density, $\sigma_{si}$ is the effective solid-fluid diameter, and $\varepsilon_{si}$ is the potential representing the interaction between surface and fluid segment. $\sigma_{si}$ and $\varepsilon_{si}$ can be determined with the Berthelot–Lorentz combining rules:

$$\{\begin{array}{c} \sigma_{si}=\frac{\sigma_{i}+\sigma_{s}}{2}\\ \varepsilon_{si}=\sqrt{\varepsilon_{i}\varepsilon_{s}} \end{array}$$ (17b)

where $\sigma_{s}$ and $\varepsilon_{s}$ are the size and potential parameters of a solid surface, respectively, and $\varepsilon_{i}$ is the potential parameters of fluid segment *i*. Here, the used potential parameters for silica are $\sigma_{s}=3.0\;\text{Å}$ , $\;\varepsilon_{s}=0.8\;kJ/mol$ , and $4{\text{πρ}}_{atom}=0.5/{\text{Å}}^{3}$ (Pinilla et al., 2005). The ePC-SAFT parameters for ILs were taken from the previous work (Ji et al., 2014), while those for CO_2_ were taken from Gross and Sadowski (2001).

For the electroneutral silica nanopore, the amount of cation and anion adsorbed per surface area should be equal. The solubility of CO_2_ in the confined IL with a neutral surface is defined by:

$$x_{CO_{2}}=\frac{{\bar{\rho }}_{CO_{2}}}{{\bar{\rho }}_{CO_{2}}+{\bar{\rho }}_{IL}}$$ (18)

where ${\bar{\rho }}_{IL}={\bar{\rho }}_{IL-cation}={\bar{\rho }}_{IL-anion}$ , according to the charge neutrality condition.

## 3 Results and discussion

### 3.1 Efficiency of the General Scheme Combined With Anderson Mixing

Calculating the density profile of [C_6_mim] [Tf_2_N]-CO_2_ confined in electronic neutral silica pore with different structures at 333 K and 16.1 bar was selected as an example here to demonstrate the performance of the general scheme combined with the Anderson mixing, and the calculation efficiency was compared with the general scheme only using the Picard iterations. In the calculation, the width of the slit-shaped pore and the diameters for the cylindrical pore and spherical cavity are 5 nm. We used 120 collocation points to represent the density profile inside the slit-shaped pore (functional derivatives only need to be calculated in half of the collocation points due to the symmetry of the density profile), while 60 collocation points were used for the cylindrical pore and spherical cavity. The relaxing parameters used in all these three cases are 0.001.

**FIGURE 2:**
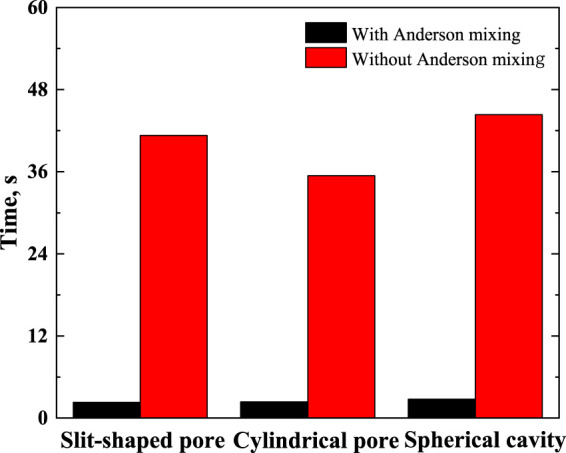
Time required for calculating equilibrium density profile (not including the time for calculating the matrices required in the Chebyshev pseudo-spectral collocation method).

The calculations were performed on a computer with an AMD core Ryzen 7 PRO 4750U and X64 processor. The version of the compiler is Matlab 2018b. Figure 2 compares the time required for calculations.

As illustrated in Figure 2, using the Anderson mixing can improve the calculation efficiency significantly. The time needed for calculating the matrices required in the Chebyshev pseudo-spectral collocation method is listed in Table 1.

**TABLE 1:** Time needed for calculating the matrices required in the Chebyshev pseudo-spectral collocation method.

	Slit-shaped pore	Cylindrical pore	Spherical cavity
Time, s	0.19	39.05	0.11

As listed in Table 1, the time required for calculating the matrices for the slit-shaped pore and spherical cavity can be ignored. However, the time for the cylindrical pore is pronounced. The reason is that too much time is used for evaluating the Legendre complete elliptic integral of the first and second kinds. This may be a problem in massive ePC-SAFT-DFT calculations, for example, adjusting the model parameters from experimental data, in which the equilibrium density profile needs to be solved multiple times.

However, for systems at the same temperature and pore diameter (cylindrical pore), the elements of these matrices are the same (Sun et al., 2021). Therefore, for the sake of saving computation time, when modeling systems confined in the cylindrical pore, these matrices can be used repeatedly for the systems at the same temperature and pore diameter.

### 3.2 Model CO_2_ Solubilities of Confined Ionic Liquids

Based on the efficient algorithm discussed above, ePC-SAFT-DFT can be used in a wide range of IL systems to obtain results efficiently. In this work, several [C_n_mim]-based IL-CO_2_ systems confined in silica nanopore were selected to investigate the effects of temperature, pressure, IL ions, as well as the size and shape of the pore. As the nanopores of different silica materials have been roughly assumed as slit-like (Li et al., 2005; Yang and Yue, 2007), cylindrical (Kresge et al., 1992; Beck et al., 1992; Maddox et al., 1997), and spherical (Nandiyanto et al., 2009) in the previous theoretical work, in this work, these three pore-shape models were adopted.

#### 3.2.1 General View of CO_2_ Confined In Silica Nanopores

**FIGURE 3:**
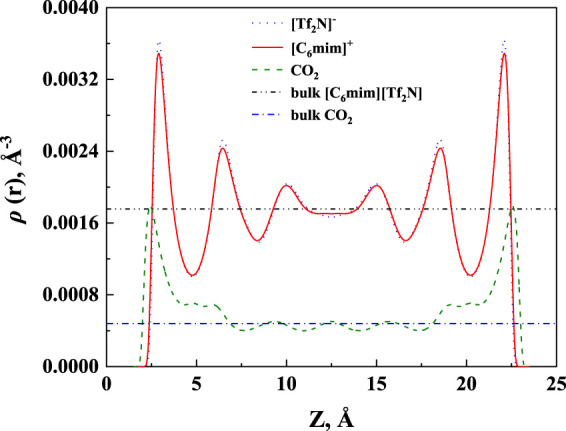
The calculated density profiles of [C_6_mim] [Tf_2_N]-CO_2_ system confined in 25 Å slit silica pore at 10 bar and 323.15 K.

The calculated density profile of [C_6_mim] [Tf_2_N]-CO_2_ confined in 25 Å slit silica pore at 10 bar and 323.15 K is presented in Figure 3.

As pointed out by Ho et al. (2013), two competitive mechanisms affect the solubility in a nanopore: One is the adsorption near the pore surface, and the other is the absorption inside the IL. These can be seen in the density profile of CO_2_. According to the results illustrated in Figure 3, the peak in the density profile near the surface of nanopores corresponds to the first mechanism, while in the middle of the nanopore, the density profile of CO_2_ tends to the bulk density, which is the reflection of the second mechanism. The absorption enhancement in the confined ILs is mainly contributed by the first mechanism. In this work, the additional solubility *x* ^*a*^ is used to identify the absorption enhancement:

$$x^{a}=x_{confined}-x_{bulk}$$ (19)

#### 3.2.2 The Influence of Temperature

**FIGURE 4:**
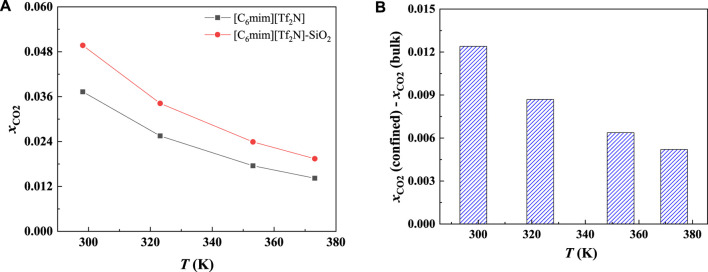
Calculated CO_2_ solubility **(A)** and additional solubilities **(B)** of ILs confined in 25 Å slit-shaped pore at 1 bar and different temperatures.

In general, with the temperature increase, the CO_2_ solubility in the bulk ILs will decrease. According to our calculation results, CO_2_ solubility in [C_6_mim] [Tf_2_N] confined in slit-shaped pore also decreases with the increase in temperature. This is consistent with the observation by Mirzaei et al. (2017). Typical examples are shown in Figures 3A and 4A. However, with the increase in temperature, the solubility of CO_2_ in [C_6_mim] [Tf_2_N] confined in SiO_2_ decreases greater than that of the bulk IL. For example, at 1 bar, when the temperature increase from 298.15 K to 373.15 K, the solubility of CO_2_ in the bulk [C_6_mim] [Tf_2_N] decreases by about 0.02 mol CO_2_/mol IL. Under the same condition, the calculated solubilities of CO_2_ in the confined [C_6_mim] [Tf_2_N] reduce by about 0.03 mol CO_2_/mol IL. It indicates that confined ILs may make it easier to desorb CO_2_.

**FIGURE 5:**
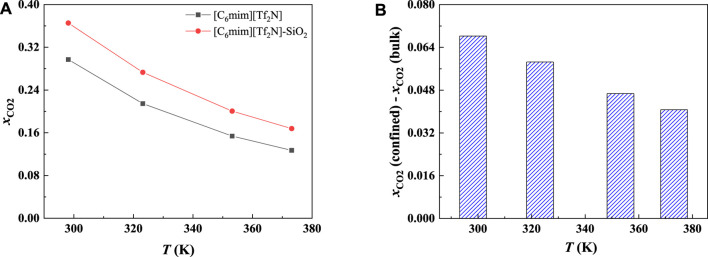
Calculated CO_2_ solubility **(A)** and additional solubilities **(B)** of ILs confined in 25 Å slit-shaped pore at 10 bar and different temperatures.

**FIGURE 6:**
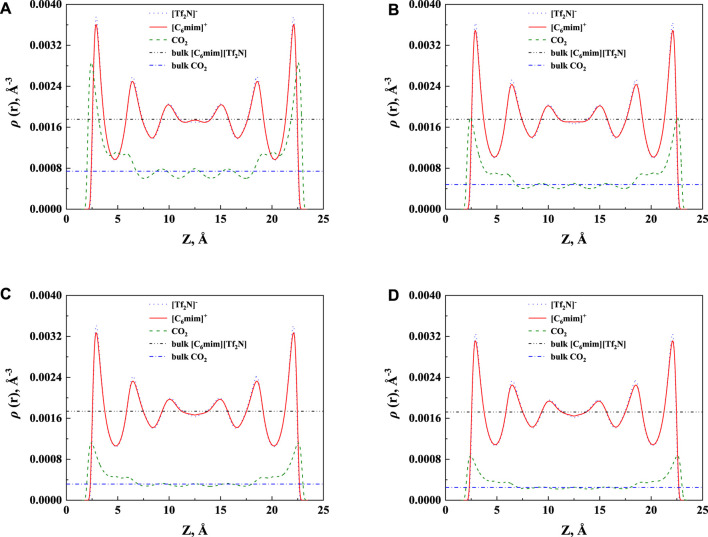
The calculated density profiles of [C_6_mim] [Tf_2_N]-CO_2_ confined in 25 Å slit silica pore at 10 bar and **(A)** 298.15 K, **(B)** 323.15 K, **(C)** 353.15 K, and **(D)** 373.15 K.

**FIGURE 7:**
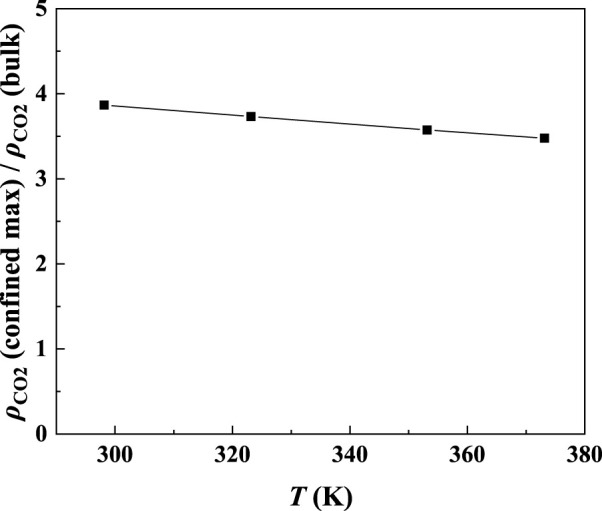
Ratios of the maximum density of CO_2_ in the confined [C_6_mim] [Tf_2_N] to its bulk density in [C_6_mim] [Tf_2_N].

In order to study the effect of temperature on the absorption enhancement effect, we analyzed the relation between the additional solubility and temperatures. As illustrated in Figures 4B and 5B, the additional solubility decreases with increasing temperature. In order to interpret this, the calculated density profiles of [C_6_mim][Tf_2_N]-CO_2_ confined in 25 Å slit silica pore at 10 bar and different temperatures are illustrated in Figure 6. To quantitatively describe the effect of the first mechanism, the ratios of the CO_2_ density at the first peak near the pore surface (which is also the maximum density inside the nanopore) to its bulk density in ILs were calculated as illustrated in Figure 7. According to the results shown in Figure 7, the ratio decreases with the increase in the temperature when the pressure keeps constant, and consequently, the additional solubility decreases. The same phenomenon can be observed at other pressures and in other pore structures, as listed in Supplementary Table S1.

#### 3.2.3 The Influence of Pressure

**FIGURE 8:**
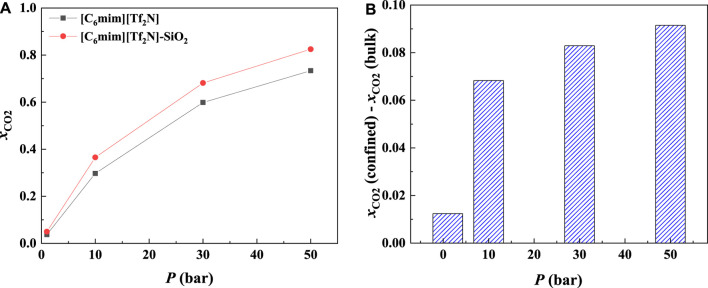
Calculated CO_2_ solubility **(A)** and additional solubilities **(B)** of confined ILs in 25 Å slit-shaped pore at 298.15 K and different pressures.

Most commercial ILs are physical absorbents for CO_2_. In general, the CO_2_ solubility in ILs will increase significantly with increasing pressure. Figure 8A illustrates the CO_2_ solubility at 298.15 K and different pressures in the 25 Å slit-shaped pore. From Figure 8B, The CO_2_ solubilities in confined ILs also increase with the increase in the pressure. In addition, the increase in CO_2_ solubility in the confined [C_6_mim] [Tf_2_N] is more significant than that of the bulk [C_6_mim] [Tf_2_N] under the same condition. For instance, when the pressure increases from 1 to 50 bar, the solubility of CO_2_ in the confined [C_6_mim] [Tf_2_N] increases by about 0.78 mol CO_2_/mol IL, while that in bulk [C_6_mim] [Tf_2_N] increases by about 0.70 mol CO_2_/mol IL.

**FIGURE 9:**
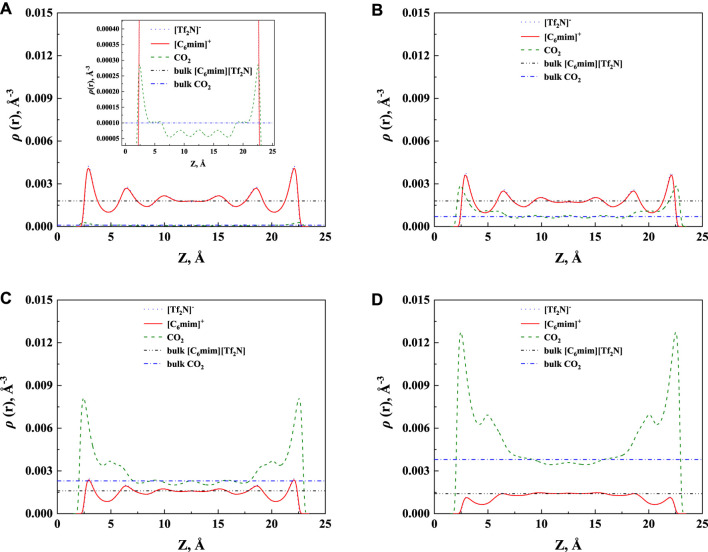
Calculated density profiles of [C_6_mim] [Tf_2_N]-CO_2_ system confined in 25 Å slit silica pore at 298.15 K and **(A)** 1 bar, **(B)** 10 bar, **(C)** 30 bar, and **(D)** 50 bar.

The additional solubility also increases with increasing pressure, as illustrated in Figure 8B. To have a deep insight into this observation, the density profiles of [C_6_mim] [Tf_2_N]-CO_2_ confined in 25 Å slit silica pore at 298.15 K and different pressures illustrated in Figure 9 were taken as one example for the detailed analysis. According to the results shown in Figure 9, with the increase in pressure, the adsorption of CO_2_ near the pore surface increases, while the adsorption of IL ions near the pore surface decreases. This can be interpreted that at high pressure, a large amount of CO_2_ inside the nanopore leads to a stronger competitive adsorption capacity of CO_2_ in the pore surface than that of IL ions. Consequently, the additional solubility increases with the increase in pressure. The same phenomenon can be observed under other conditions, as listed in Supplementary Table S1.

#### 3.2.4 The Influence of Pore Size and Shape

**FIGURE 10:**
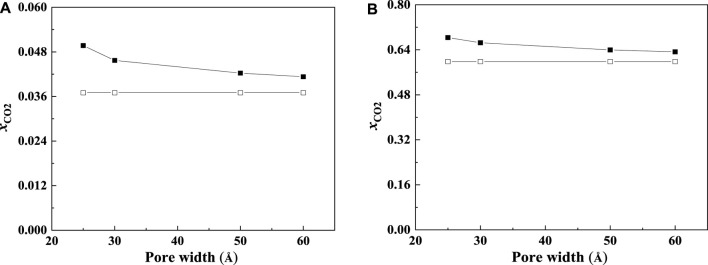
Calculated CO_2_ solubility of [C_6_mim] [Tf_2_N] at 298.15 K and 1 bar **(A)** and 30 bar **(B)** in slit-shaped pores with different widths (solid symbols, data for confined ILs; open symbols, data for bulk ILs.).

The CO_2_ solubilities of confined ILs will also be affected by the pore size and shape. According to the calculation results, the adsorption will be enhanced more significantly in the smaller nanopore, which is consistent with the results of Shi and Luebke (2013). Two typical examples are shown in Figure 10. As illustrated in Figure 10, the solubilities of CO_2_ increase with the decrease in the slit-shaped pore.

**FIGURE 11:**
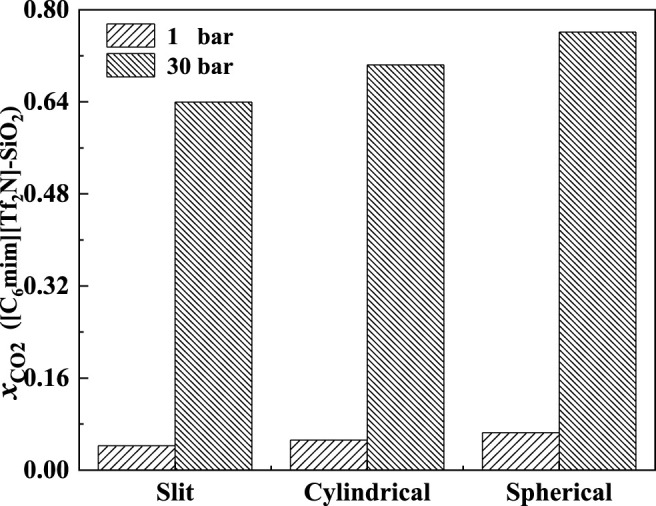
Calculated CO_2_ solubility of [C_6_mim] [Tf_2_N] confined in different-shaped pores at 298.15 K and 1 and 30 bar (the width of slit-shaped pore and the diameter of cylindrical pore and spherical cavity are all 5 nm).

**FIGURE 12:**
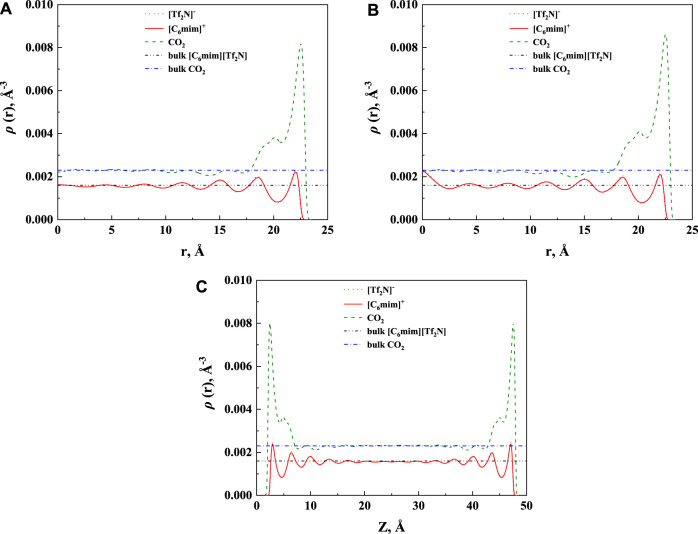
Calculated density profiles [C_6_mim] [Tf_2_N]-CO_2_ confined in 50 Å **(A)** cylindrical, **(B)** spherical, and **(C)** slit-shaped silica pore at 298.15 K and 30 bar.

The effect of the shape of nanopores was also investigated. According to our calculation results, for nanopores with the same size (i.e., the width for slit-shaped pore and the diameter for cylindrical pore and spherical cavity), ILs confined in the spherical cavity have the highest CO_2_ solubilities, while those confined in the slit-shaped pore have the lowest. Typical examples are illustrated in Figure 11. This indicates that using supported material with spherical nanopores may lead to better absorption capacity. The calculated density profiles of the three shapes at 298.15 K and 30 bar are presented in Figure 12. It shows that the pore shape affects the density profile near the surface of the pore significantly. For IL ions, the density at the first peak follows the trend that ρ (slit-shaped) > ρ (cylindrical) > ρ (spherical), while for CO_2_, the opposite trend can be observed. This is because the inward curved surfaces impede the accumulation of large molecules (e.g., IL ions) near the surface. Therefore, under the same situation, the CO_2_ solubilities (*x*) follow the trend that *x* (spherical) > *x* (cylindrical) > *x* (slit-shaped).

#### 3.2.5 The Influence of Cation

In most cases, the IL-cations are chain-like substances. Therefore, in this part, the effect of alkyl-chain length was studied. Following this, ePC-SAFT-DFT was used to model the CO_2_ solubilities of [C_4_mim][Tf_2_N], [C_6_mim] [Tf_2_N], and [C_8_mim] [Tf_2_N] inside silica nanopores at different temperatures, pressures, pore widths, and pore shapes. The calculated results are listed in Supplementary Table S1.

**FIGURE 13:**
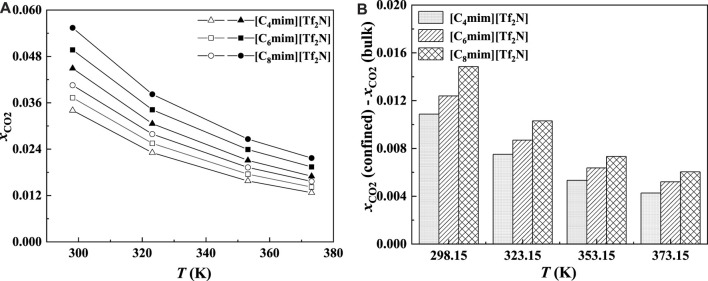
Calculated CO_2_ solubility **(A)** and additional solubilities **(B)** of ILs confined in 25 Å slit-shaped pore at 1 bar and different temperatures. **(A)** Solid symbols, data for confined ILs; open symbols, data for bulk ILs.

**FIGURE 14:**
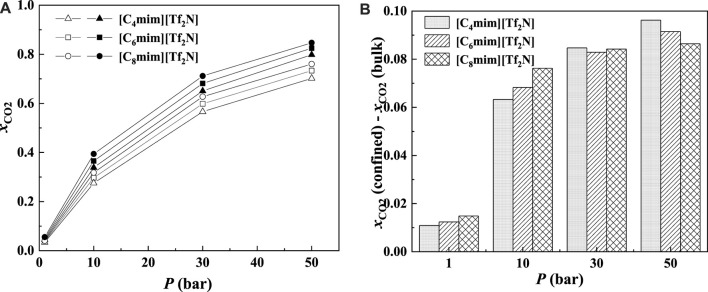
Calculated CO_2_ solubility **(A)** and additional solubilities **(B)** of ILs confined in 25 Å slit-shaped pore at 298.15 K and different pressures. **(A)** Solid symbols, data for confined ILs; open symbols, data for bulk ILs.

As illustrated in Figures 13A and 14A, for the ILs in the same homologous series, the CO_2_ solubilities in the confined ILs increase with increasing alkyl-chain length in cation, which is the same as that in the bulk ILs. According to Figure 13B and Figure 14B, at low pressures, the additional solubility also increases with the increase in the alkyl-chain length. However, this is not true at high pressures, as illustrated in Figure 14B. This indicates that the absorption enhancement of ILs with longer alkyl-chain decreases faster with the increasing pressure than the ILs with shorter alkyl-chain.

**FIGURE 15:**
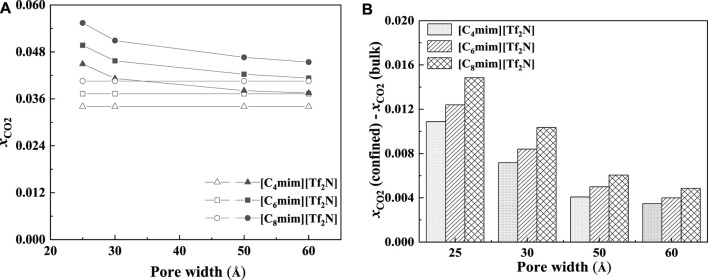
Calculated CO_2_ solubility **(A)** and additional solubilities **(B)** of ILs confined in slit-shaped pore with different widths at 298.15 K and 1 bar. **(A)** Solid symbols, data for confined ILs; open symbols, data for bulk ILs.

**FIGURE 16:**
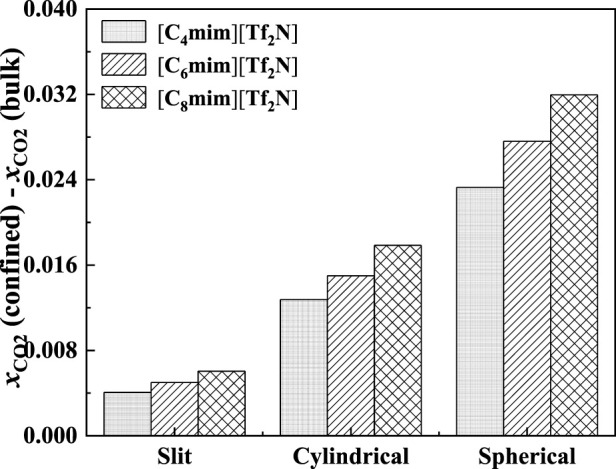
Calculated additional solubilities of ILs confined in different shaped pores at 298.15 K and 1 bar in 50 Å. Solid symbols, data for confined ILs; open symbols, data for bulk ILs.

As demonstrated in Figure 15, with the increase in pore width, the additional solubility decreases, especially for the IL with longer alkyl-chain length in the IL cation. The additional solubilities of confined ILs in different pore shapes are illustrated in Figure 16. In the spherical cavity, the additional solubilities increase more significantly with increasing alkyl-chain length in IL cation than that in the slit-shaped and cylindrical pores.

#### 3.2.6 The Influence of Anion

**FIGURE 17:**
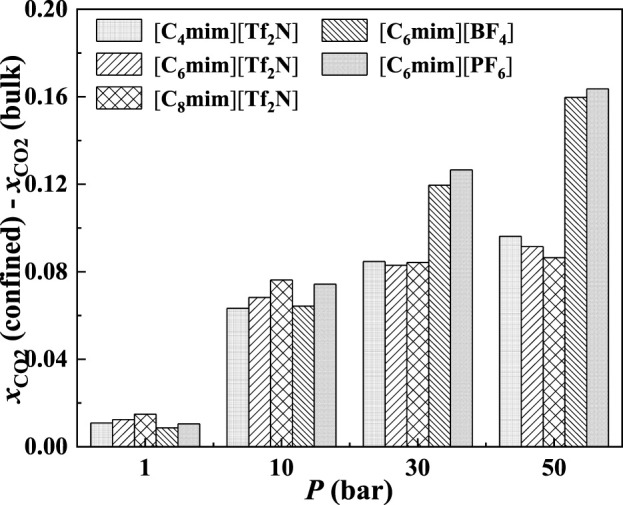
Calculated additional solubilities of ILs confined in 25 Å slit-shaped pore at 298.15 K and different pressures.

In the bulk phase, the IL anion affects the CO_2_ solubilities more significantly than IL cation (Anthony et al., 2005). According to Figure 17, the IL anion also has a more significant effect than IL cation in the absorption enhancement. From 1 to 50 bar, the deviation of the calculated additional solubility between [C_4_mim] [Tf_2_N] and [C_8_mim] [Tf_2_N] changes from 0.003 to 0.009, while for [C_6_mim] [Tf_2_N] and [C_6_mim] [PF_6_], the deviation of the calculated additional solubility changes from 0.002 to 0.071 from 1 to 50 bar.

**FIGURE 18:**
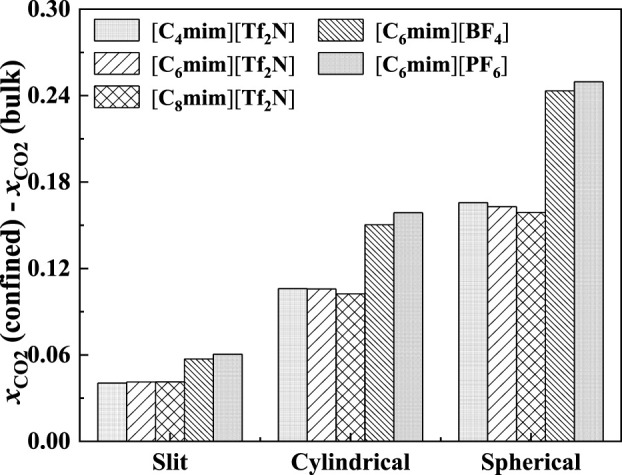
Calculated additional solubilities of ILs in different-shaped pores at 298.15 K and 30 bar (the width of slit-shaped pore and the diameter of cylindrical pore and spherical cavity are all 5 nm).

In Figure 18, the calculated additional solubilities of several confined ILs in different pore shapes are presented. The absorption enhancement of anion is more significant in the spherical cavity as shown in Figure 18. For example, in the slit-shaped pore at 30 bar, the deviation of the calculated additional solubilities between [C_4_mim] [Tf_2_N] and [C_8_mim] [Tf_2_N] is 0.001, and that between [C_6_mim] [Tf_2_N] and [C_6_mim] [PF_6_] is 0.007. While in the spherical pore, these two values are 0.007 and 0.086. In addition, for [C_6_mim] [BF_4_] and [C_6_mim] [PF_6_], the additional solubilities are considerably higher than those of [C_6_mim] [Tf_2_N] in the spherical cavity at high pressures. This indicates that changing the IL anion of ILs confined in the spherical cavity may be promising to obtain a large absorption enhancement.

Up to here of this section, the effects of temperature, pressure, IL ions, as well as the size and shape of pores on confined CO_2_ solubilities were investigated. In order to further improve model performance toward practical applications, the ePC-SAFT-DFT model will be combined with different pore models (pore shape and pore size distribution) with the parameters adjusted from experimental data in our future work based on the newly measured experimental data.

## 4 Conclusion

In this work, the Chebyshev pseudo-spectral collocation method was combined with the Anderson mixing algorithm to further accelerate the ePC-SAFT-DFT calculation. The results show that the computing time can be significantly reduced with the Anderson mixing algorithm. This makes the ePC-SAFT-DFT model more effective in screening promising ILs. However, calculating matrices used in the Chebyshev pseudo-spectral collocation method for the cylindrical pores requires a certain computing time, while in a massive computation, these matrices can be reused to save computation time.

Using the ePC-SAF-DFT model, the CO_2_ solubilities of ionic liquid confined *in silica* nanopores were studied. The results show that the CO_2_ solubilities of confined ILs are always higher than that in bulk ILs under the same condition, and the absorption enhancement effect will be significantly affected by pressure, pore widths, pore shapes, and IL anion. Based on the simulation results, to obtain high CO_2_ solubilities in silica-confined ILs, a better option is to use silica material with a narrow spherical pore. In addition, the IL anion should be selected specifically considering that the category of IL anion has a significant impact on the absorption enhancement effect.

## Data Availability

The original contributions presented in the study are included in the article/Supplementary Materials, further inquiries can be directed to the corresponding authors.
