# Supplementary material for: Accelerate the Electrolyte Perturbed-Chain Statistical Associating Fluid Theory–Density Functional Theory Calculation With the Chebyshev Pseudo-Spectral Collocation Method. Part II. Spherical Geometry and Anderson Mixing
Source: Front Chem. 2022 Jan 24;9:801551. doi: 10.3389/fchem.2021.801551 (PMC8818718; doi:10.3389/fchem.2021.801551)
Supplement: Supplementary file 1 [file Table1.DOCX]

Supplementary Material

# Supplementary Data

Table S1. The calculation results

| **Ionic liquid** | **T (K)** | **P (bar)** | **width (Å)** | **shape** | ***x_CO2_* (bulk)** | ***x_CO2_* (confined)** | ***x^a^*** |
| --- | --- | --- | --- | --- | --- | --- | --- |
| [C_4_mim][NTf_2_] | 298.15 | 1 | 25 | slit | 0.034 | 0.045 | 0.011 |
| [C_4_mim][NTf_2_] | 298.15 | 1 | 30 | slit | 0.034 | 0.041 | 0.007 |
| [C_4_mim][NTf_2_] | 298.15 | 1 | 50 | cylindrical | 0.034 | 0.047 | 0.013 |
| [C_4_mim][NTf_2_] | 298.15 | 1 | 50 | slit | 0.034 | 0.038 | 0.004 |
| [C_4_mim][NTf_2_] | 298.15 | 1 | 50 | spherical | 0.034 | 0.057 | 0.023 |
| [C_4_mim][NTf_2_] | 298.15 | 1 | 60 | slit | 0.034 | 0.038 | 0.004 |
| [C_4_mim][NTf_2_] | 298.15 | 10 | 25 | slit | 0.275 | 0.339 | 0.064 |
| [C_4_mim][NTf_2_] | 298.15 | 10 | 30 | slit | 0.275 | 0.321 | 0.046 |
| [C_4_mim][NTf_2_] | 298.15 | 10 | 50 | slit | 0.275 | 0.303 | 0.028 |
| [C_4_mim][NTf_2_] | 298.15 | 10 | 60 | slit | 0.275 | 0.298 | 0.023 |
| [C_4_mim][NTf_2_] | 298.15 | 30 | 25 | slit | 0.566 | 0.651 | 0.085 |
| [C_4_mim][NTf_2_] | 298.15 | 30 | 30 | slit | 0.566 | 0.632 | 0.066 |
| [C_4_mim][NTf_2_] | 298.15 | 30 | 50 | cylindrical | 0.566 | 0.672 | 0.106 |
| [C_4_mim][NTf_2_] | 298.15 | 30 | 50 | slit | 0.566 | 0.607 | 0.041 |
| [C_4_mim][NTf_2_] | 298.15 | 30 | 50 | spherical | 0.566 | 0.732 | 0.166 |
| [C_4_mim][NTf_2_] | 298.15 | 30 | 60 | slit | 0.566 | 0.600 | 0.034 |
| [C_4_mim][NTf_2_] | 298.15 | 50 | 25 | slit | 0.702 | 0.798 | 0.096 |
| [C_4_mim][NTf_2_] | 298.15 | 50 | 30 | slit | 0.702 | 0.782 | 0.08 |
| [C_4_mim][NTf_2_] | 298.15 | 50 | 50 | slit | 0.702 | 0.751 | 0.049 |
| [C_4_mim][NTf_2_] | 298.15 | 50 | 60 | slit | 0.702 | 0.742 | 0.04 |
| [C_4_mim][NTf_2_] | 323.15 | 1 | 25 | slit | 0.023 | 0.031 | 0.008 |
| [C_4_mim][NTf_2_] | 353.15 | 1 | 25 | slit | 0.016 | 0.021 | 0.005 |
| [C_4_mim][NTf_2_] | 373.15 | 1 | 25 | slit | 0.013 | 0.017 | 0.004 |
| [C_6_mim][BF_4_] | 298.15 | 1 | 25 | slit | 0.020 | 0.029 | 0.009 |
| [C_6_mim][BF_4_] | 298.15 | 1 | 30 | slit | 0.020 | 0.026 | 0.006 |
| [C_6_mim][BF_4_] | 298.15 | 1 | 50 | cylindrical | 0.020 | 0.031 | 0.011 |
| [C_6_mim][BF_4_] | 298.15 | 1 | 50 | slit | 0.020 | 0.024 | 0.004 |
| [C_6_mim][BF_4_] | 298.15 | 1 | 50 | spherical | 0.020 | 0.040 | 0.02 |
| [C_6_mim][BF_4_] | 298.15 | 1 | 60 | slit | 0.020 | 0.023 | 0.003 |
| [C_6_mim][BF_4_] | 298.15 | 10 | 25 | slit | 0.177 | 0.241 | 0.064 |
| [C_6_mim][BF_4_] | 298.15 | 10 | 30 | slit | 0.177 | 0.224 | 0.047 |
| [C_6_mim][BF_4_] | 298.15 | 10 | 50 | slit | 0.177 | 0.204 | 0.027 |
| [C_6_mim][BF_4_] | 298.15 | 10 | 60 | slit | 0.177 | 0.200 | 0.023 |
| [C_6_mim][BF_4_] | 298.15 | 30 | 25 | slit | 0.408 | 0.527 | 0.119 |
| [C_6_mim][BF_4_] | 298.15 | 30 | 30 | slit | 0.408 | 0.503 | 0.095 |
| [C_6_mim][BF_4_] | 298.15 | 30 | 50 | cylindrical | 0.408 | 0.558 | 0.150 |
| [C_6_mim][BF_4_] | 298.15 | 30 | 50 | slit | 0.408 | 0.465 | 0.057 |
| [C_6_mim][BF_4_] | 298.15 | 30 | 50 | spherical | 0.408 | 0.651 | 0.243 |
| [C_6_mim][BF_4_] | 298.15 | 30 | 60 | slit | 0.408 | 0.456 | 0.048 |
| [C_6_mim][BF_4_] | 298.15 | 50 | 25 | slit | 0.540 | 0.700 | 0.16 |
| [C_6_mim][BF_4_] | 298.15 | 50 | 30 | slit | 0.540 | 0.674 | 0.134 |
| [C_6_mim][BF_4_] | 298.15 | 50 | 50 | slit | 0.540 | 0.623 | 0.083 |
| [C_6_mim][BF_4_] | 298.15 | 50 | 60 | slit | 0.540 | 0.610 | 0.07 |
| [C_6_mim][BF_4_] | 323.15 | 1 | 25 | slit | 0.014 | 0.020 | 0.006 |
| [C_6_mim][BF_4_] | 353.15 | 1 | 25 | slit | 0.009 | 0.014 | 0.005 |
| [C_6_mim][BF_4_] | 373.15 | 1 | 25 | slit | 0.008 | 0.011 | 0.003 |
| [C_6_mim][NTf_2_] | 298.15 | 1 | 25 | slit | 0.037 | 0.050 | 0.013 |
| [C_6_mim][NTf_2_] | 298.15 | 1 | 30 | slit | 0.037 | 0.046 | 0.009 |
| [C_6_mim][NTf_2_] | 298.15 | 1 | 50 | cylindrical | 0.037 | 0.052 | 0.015 |
| [C_6_mim][NTf_2_] | 298.15 | 1 | 50 | slit | 0.037 | 0.042 | 0.005 |
| [C_6_mim][NTf_2_] | 298.15 | 1 | 50 | spherical | 0.037 | 0.065 | 0.028 |
| [C_6_mim][NTf_2_] | 298.15 | 1 | 60 | slit | 0.037 | 0.041 | 0.004 |
| [C_6_mim][NTf_2_] | 298.15 | 10 | 25 | slit | 0.297 | 0.365 | 0.068 |
| [C_6_mim][NTf_2_] | 298.15 | 10 | 30 | slit | 0.297 | 0.349 | 0.052 |
| [C_6_mim][NTf_2_] | 298.15 | 10 | 50 | slit | 0.297 | 0.328 | 0.031 |
| [C_6_mim][NTf_2_] | 298.15 | 10 | 60 | slit | 0.297 | 0.322 | 0.025 |
| [C_6_mim][NTf_2_] | 298.15 | 30 | 25 | slit | 0.598 | 0.681 | 0.083 |
| [C_6_mim][NTf_2_] | 298.15 | 30 | 30 | slit | 0.598 | 0.665 | 0.067 |
| [C_6_mim][NTf_2_] | 298.15 | 30 | 50 | cylindrical | 0.598 | 0.704 | 0.106 |
| [C_6_mim][NTf_2_] | 298.15 | 30 | 50 | slit | 0.598 | 0.640 | 0.042 |
| [C_6_mim][NTf_2_] | 298.15 | 30 | 50 | spherical | 0.598 | 0.761 | 0.163 |
| [C_6_mim][NTf_2_] | 298.15 | 30 | 60 | slit | 0.598 | 0.633 | 0.035 |
| [C_6_mim][NTf_2_] | 298.15 | 50 | 25 | slit | 0.733 | 0.825 | 0.092 |
| [C_6_mim][NTf_2_] | 298.15 | 50 | 30 | slit | 0.733 | 0.810 | 0.077 |
| [C_6_mim][NTf_2_] | 298.15 | 50 | 50 | slit | 0.733 | 0.781 | 0.048 |
| [C_6_mim][NTf_2_] | 298.15 | 50 | 60 | slit | 0.733 | 0.772 | 0.039 |
| [C_6_mim][NTf_2_] | 323.15 | 1 | 25 | slit | 0.025 | 0.034 | 0.009 |
| [C_6_mim][NTf_2_] | 353.15 | 1 | 25 | slit | 0.018 | 0.024 | 0.006 |
| [C_6_mim][NTf_2_] | 373.15 | 1 | 25 | slit | 0.014 | 0.019 | 0.005 |
| [C_6_mim][PF_6_] | 298.15 | 1 | 25 | slit | 0.022 | 0.033 | 0.011 |
| [C_6_mim][PF_6_] | 298.15 | 1 | 30 | slit | 0.022 | 0.029 | 0.007 |
| [C_6_mim][PF_6_] | 298.15 | 1 | 50 | cylindrical | 0.022 | 0.035 | 0.013 |
| [C_6_mim][PF_6_] | 298.15 | 1 | 50 | slit | 0.022 | 0.026 | 0.004 |
| [C_6_mim][PF_6_] | 298.15 | 1 | 50 | spherical | 0.022 | 0.045 | 0.023 |
| [C_6_mim][PF_6_] | 298.15 | 1 | 60 | slit | 0.022 | 0.025 | 0.003 |
| [C_6_mim][PF_6_] | 298.15 | 10 | 25 | slit | 0.192 | 0.267 | 0.075 |
| [C_6_mim][PF_6_] | 298.15 | 10 | 30 | slit | 0.192 | 0.244 | 0.052 |
| [C_6_mim][PF_6_] | 298.15 | 10 | 50 | slit | 0.192 | 0.223 | 0.031 |
| [C_6_mim][PF_6_] | 298.15 | 10 | 60 | slit | 0.192 | 0.218 | 0.026 |
| [C_6_mim][PF_6_] | 298.15 | 30 | 25 | slit | 0.439 | 0.565 | 0.126 |
| [C_6_mim][PF_6_] | 298.15 | 30 | 30 | slit | 0.439 | 0.538 | 0.099 |
| [C_6_mim][PF_6_] | 298.15 | 30 | 50 | cylindrical | 0.439 | 0.598 | 0.159 |
| [C_6_mim][PF_6_] | 298.15 | 30 | 50 | slit | 0.439 | 0.499 | 0.06 |
| [C_6_mim][PF_6_] | 298.15 | 30 | 50 | spherical | 0.439 | 0.688 | 0.249 |
| [C_6_mim][PF_6_] | 298.15 | 30 | 60 | slit | 0.439 | 0.489 | 0.05 |
| [C_6_mim][PF_6_] | 298.15 | 50 | 25 | slit | 0.576 | 0.739 | 0.163 |
| [C_6_mim][PF_6_] | 298.15 | 50 | 30 | slit | 0.576 | 0.713 | 0.137 |
| [C_6_mim][PF_6_] | 298.15 | 50 | 50 | slit | 0.576 | 0.663 | 0.087 |
| [C_6_mim][PF_6_] | 298.15 | 50 | 60 | slit | 0.576 | 0.649 | 0.073 |
| [C_6_mim][PF_6_] | 323.15 | 1 | 25 | slit | 0.015 | 0.022 | 0.007 |
| [C_6_mim][PF_6_] | 353.15 | 1 | 25 | slit | 0.010 | 0.016 | 0.006 |
| [C_6_mim][PF_6_] | 373.15 | 1 | 25 | slit | 0.008 | 0.013 | 0.005 |
| [C_8_mim][NTf_2_] | 298.15 | 1 | 25 | slit | 0.041 | 0.055 | 0.014 |
| [C_8_mim][NTf_2_] | 298.15 | 1 | 30 | slit | 0.041 | 0.051 | 0.01 |
| [C_8_mim][NTf_2_] | 298.15 | 1 | 50 | cylindrical | 0.041 | 0.058 | 0.017 |
| [C_8_mim][NTf_2_] | 298.15 | 1 | 50 | slit | 0.041 | 0.047 | 0.006 |
| [C_8_mim][NTf_2_] | 298.15 | 1 | 50 | spherical | 0.041 | 0.073 | 0.032 |
| [C_8_mim][NTf_2_] | 298.15 | 1 | 60 | slit | 0.041 | 0.045 | 0.004 |
| [C_8_mim][NTf_2_] | 298.15 | 10 | 25 | slit | 0.318 | 0.394 | 0.076 |
| [C_8_mim][NTf_2_] | 298.15 | 10 | 30 | slit | 0.318 | 0.375 | 0.057 |
| [C_8_mim][NTf_2_] | 298.15 | 10 | 50 | slit | 0.318 | 0.352 | 0.034 |
| [C_8_mim][NTf_2_] | 298.15 | 10 | 60 | slit | 0.318 | 0.346 | 0.028 |
| [C_8_mim][NTf_2_] | 298.15 | 30 | 25 | slit | 0.627 | 0.712 | 0.085 |
| [C_8_mim][NTf_2_] | 298.15 | 30 | 30 | slit | 0.627 | 0.696 | 0.069 |
| [C_8_mim][NTf_2_] | 298.15 | 30 | 50 | cylindrical | 0.627 | 0.730 | 0.103 |
| [C_8_mim][NTf_2_] | 298.15 | 30 | 50 | slit | 0.627 | 0.669 | 0.042 |
| [C_8_mim][NTf_2_] | 298.15 | 30 | 50 | spherical | 0.627 | 0.786 | 0.159 |
| [C_8_mim][NTf_2_] | 298.15 | 30 | 60 | slit | 0.627 | 0.662 | 0.035 |
| [C_8_mim][NTf_2_] | 298.15 | 50 | 25 | slit | 0.760 | 0.847 | 0.087 |
| [C_8_mim][NTf_2_] | 298.15 | 50 | 30 | slit | 0.760 | 0.834 | 0.074 |
| [C_8_mim][NTf_2_] | 298.15 | 50 | 50 | slit | 0.760 | 0.806 | 0.046 |
| [C_8_mim][NTf_2_] | 298.15 | 50 | 60 | slit | 0.760 | 0.798 | 0.038 |
| [C_8_mim][NTf_2_] | 323.15 | 1 | 25 | slit | 0.028 | 0.038 | 0.01 |
| [C_8_mim][NTf_2_] | 353.15 | 1 | 25 | slit | 0.019 | 0.027 | 0.008 |
| [C_8_mim][NTf_2_] | 373.15 | 1 | 25 | slit | 0.016 | 0.022 | 0.006 |
